# Acylation driven by intracellular metabolites in host cells inhibits Cas9 activity used for genome editing

**DOI:** 10.1093/pnasnexus/pgac277

**Published:** 2022-12-06

**Authors:** Li Zhao, Di You, Ting Wang, Zhen-Ping Zou, Bin-Cheng Yin, Ying Zhou, Bang-Ce Ye

**Affiliations:** Laboratory of Biosystems and Microanalysis, State Key Laboratory of Bioreactor Engineering, East China University of Science and Technology, Shanghai 200237, China; Laboratory of Biosystems and Microanalysis, State Key Laboratory of Bioreactor Engineering, East China University of Science and Technology, Shanghai 200237, China; Laboratory of Biosystems and Microanalysis, State Key Laboratory of Bioreactor Engineering, East China University of Science and Technology, Shanghai 200237, China; Laboratory of Biosystems and Microanalysis, State Key Laboratory of Bioreactor Engineering, East China University of Science and Technology, Shanghai 200237, China; Laboratory of Biosystems and Microanalysis, State Key Laboratory of Bioreactor Engineering, East China University of Science and Technology, Shanghai 200237, China; Institute of Engineering Biology and Health, Collaborative Innovation Center of Yangtze River Delta Region Green Pharmaceuticals, College of Pharmaceutical Sciences, Zhejiang University of Technology, Hangzhou 310014, Zhejiang, China; Laboratory of Biosystems and Microanalysis, State Key Laboratory of Bioreactor Engineering, East China University of Science and Technology, Shanghai 200237, China; Laboratory of Biosystems and Microanalysis, State Key Laboratory of Bioreactor Engineering, East China University of Science and Technology, Shanghai 200237, China; Institute of Engineering Biology and Health, Collaborative Innovation Center of Yangtze River Delta Region Green Pharmaceuticals, College of Pharmaceutical Sciences, Zhejiang University of Technology, Hangzhou 310014, Zhejiang, China

**Keywords:** Cas9, acylation, Crispr, genome editing

## Abstract

CRISPR-Cas, the immune system of bacteria and archaea, has been widely harnessed for genome editing, including gene knockouts and knockins, single-base editing, gene activation, and silencing. However, the molecular mechanisms underlying fluctuations in the genome editing efficiency of crispr in various cells under different conditions remain poorly understood. In this work, we found that Cas9 can be ac(et)ylated by acetyl-phosphate or acyl-CoA metabolites both *in vitro* and *in vivo*. Several modifications are associated with the DNA or sgRNA binding sites. Notably, ac(et)ylation of Cas9 driven by these metabolites in host cells potently inhibited its binding and cleavage activity with the target DNA, thereby decreasing Crispr genome editing efficiency. This study provides more insights into understanding the effect of the intracellular environment on genome editing application of crispr with varying efficiency in hosts.

Significance Statement In this work, we demonstrated that ac(et)ylation of Cas enzyme driven by intracellular acetyl-phosphate or acyl-CoA metabolites in host cells inhibited its binding and cleavage activity with target nucleic acids, thereby decreasing the genome editing efficiency of CRISPR. The findings provide a promising paradigm for optimizing Cas enzymes and improving CRISPR efficiency.

## Introduction

**Fig. 1.**
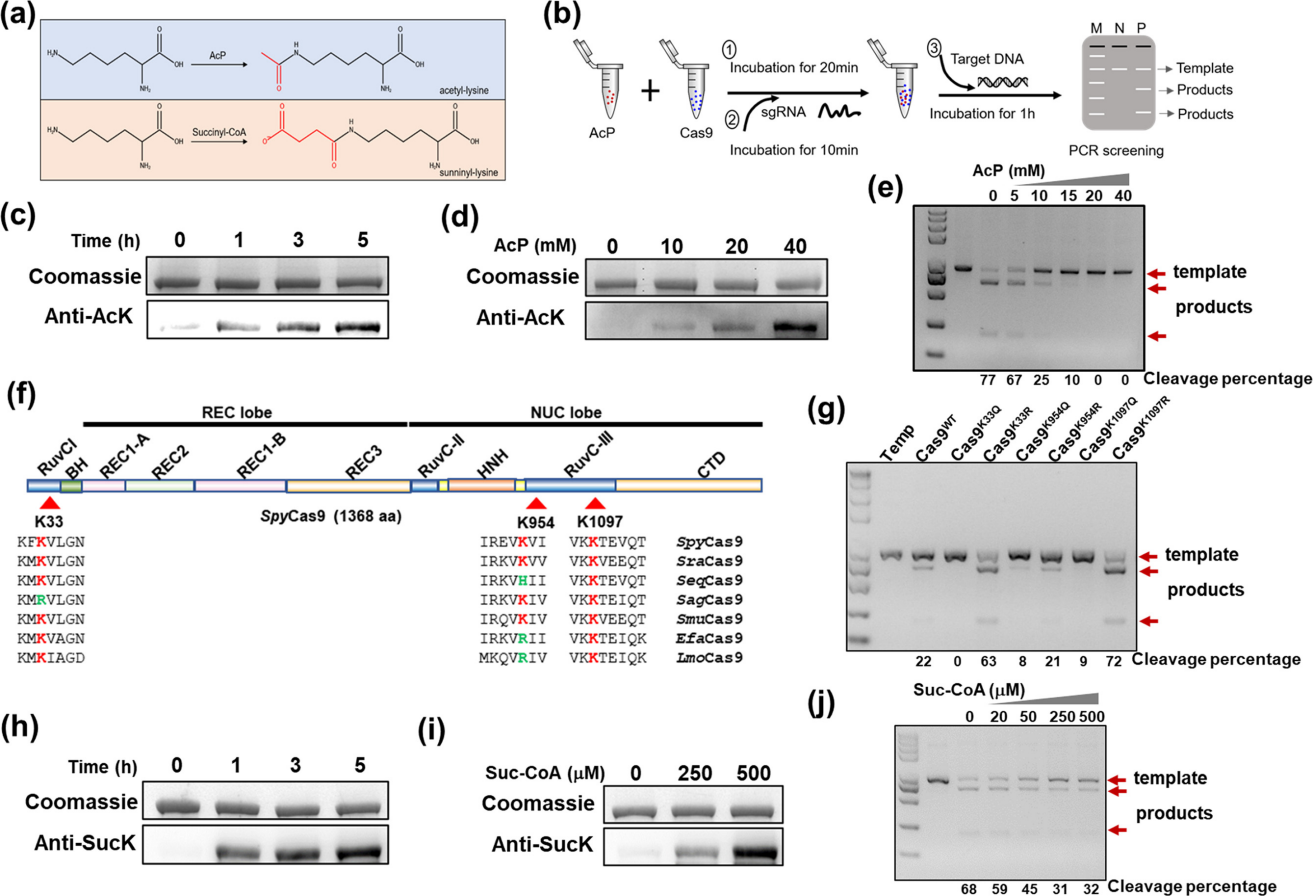
Ac(et)ylation of *Spy*Cas9 decreased the cleavage activity *in vitro*. (a) Diagram of N^ε^-Lys-ac(et)ylation of Cas9 by AcP or succinyl-CoA. (b) Diagram of the assay scheme for the acylation and cleavage experiment. (c) Western blot analysis of Cas9 incubated with AcP (10 mM) for 0, 1, 3, and 5 h at 37°C by using anti-AcK. (d) Western blot analysis of Cas9 incubated with AcP (0, 10, 20, and 40 mM) for 5 h at 37°C by using anti-AcK. (e) Cleavage inhibition assay by using Cas9 protein acetylated with increasing concentrations of AcP (0, 5, 10, 15, 20, and 40 mM). (f) Domain organization of Cas9. K33, K1097, and K954 are located at the RuvC domain. (g) Cleavage efficiency was measured using the mutated Cas9 (K33Q, K33R, K954Q, K954R, K1097Q, and K1097R). (h) Western blot analysis of Cas9 incubated with succinyl-CoA (500 μM) for 0, 1, 3, and 5 h at 37°C by using anti-SucK. (i) Western blot analysis of Cas9 incubated with succinyl-CoA (0,250, and 500 μM) for 5 h at 37°C by using anti-SucK. (j) Cleavage inhibition assay by using Cas9 protein succinylated with increasing concentrations of succinyl-CoA (0, 20, 50,250, and 500 μM).

CRISPR-Cas systems, which exist widely in bacteria and archaea, serve as adaptive immune systems against invasive nucleic acids such as phages or plasmids (1). In the past decade, sgRNA-guided Cas nuclease has been employed to develop powerful new tools for genome editing and gene regulation (2–7). Accurate control of Cas enzyme activity in host cells is important for practical genome-editing applications of this biotechnology. Many anti-CRISPR proteins (Acrs), which have evolved to evade CRISPR systems, have been shown to adjust the CRISPR-Cas activity by protein–protein interactions or by protein modification (8). Recently, Acr-mediated acetylation and ADP-ribosylation of the Cas system have been reported (9,10). AcrIF11, an ADP-ribosyltransferase, specifically modifies the N250 residue in the PAM recognition loop of type I Cas8f, thereby inhibiting its DNA binding activity and inactivating the CRISPR system (9). AcrVA5 is an acetyltransferase disabled type V Cas12a that acetylates the K635 residue in the DNA-binding interface of *Mb*Cas12a to block target DNA binding (10). Ac(et)ylation is a major posttranslational protein modification that plays important regulatory roles in enzyme activity, protein stability, protein–protein interactions, protein-DNA binding affinity, and protein localization (11). Accumulating experimental data demonstrated that protein ac(et)ylation can occur via two distinct mechanisms: enzymatic acylation, which relies on acetyltransferases, and nonenzymatic acylation (chemical acetylation), which is driven by intracellular high-energy intermediates such as acetyl-phosphate (AcP) or acyl-CoAs. In *Escherichia coli*, nonenzymatic acetylation with AcP is more global and less specific than enzymatic acetylation, and is thought to predominate in affecting protein acetylation levels (12). On the other hand, many lysine residues at the active sites are important for the binding and cleavage activities of Cas with target DNA. These observations raise the possibility that Cas9 proteins introduced into the cells may be covalently modified by nonenzymatic ac(et)ylation driven by intracellular acetyl-phosphate or acyl-CoAs (Fig. 1a), which exerts an inhibitory effect on the genome editing application of CRISPR in host cells. To test this hypothesis, we investigated the ac(et)ylation of *Spy*Cas9 and its effect on Cas9 activity for genome editing.

## Results and discussion

### Ac(et)ylation of *Spy*Cas9 decreased the cleavage activity *in vitro*

Intracellular accumulation of AcP significantly increases the level of protein acetylation in *E. coli (*12). To assess the inhibitory effects of acetylation on Cas9 activity, we first investigated whether AcP acetylates *Spy*Cas9 protein from *Streptococcus pyogenes* and affects its activity. The level of acetylation of Cas9 protein incubated with different concentrations of AcP for different periods of time was determined by western blotting, and the cleavage activity of Cas9 treated with AcP was examined by gel imaging (Fig. 1b). The results showed that the acetylation level of Cas9 gradually increased with extended incubation time (Fig. 1c) and increased AcP concentration (Fig. 1d). The cleavage inhibition assay revealed that Cas9 preincubated with AcP was compromised in its cleavage activity (Fig. 1e). Many lysine acetylation sites were identified in AcP-treated Cas9 by Nano-HPLC-MS/MS analysis, among which K33, K954, and K1097 showed obvious increases in acetylation level when treated with 40 mM AcP (Fig. S1). K1097 of Cas9 was highly conserved among Cas9 proteins from different strains (Figs. 1f and S2). Notably, K33 and K954 were not completely conserved, as some Cas9 proteins (*Sag*1Cas9 and *Sag*2Cas9) from *Streptococcus agalactiae* had arginine residues at the K33 position, and some Cas9 proteins (*Efa*Cas9, *Lmo*Cas9, and *Seq*2Cas9) from *Enterococcus faecium, Listeria monocytogenes*, and *Streptococcus equinus* had arginine or histidine residues at the K954 position. Arginine and histidine are positively charged under physiological conditions and cannot be acylated. Substituting arginine or histidine for lysine may alleviate the effects of acylation on Cas9 activity. K33, K954, and K1097 are located in the RuvC domain (Fig. 1f), and are responsible for dsDNA cleavage. K33 and K1097 both interact with the sgRNA at G81 and U66/C67 directly (13) and K954 interacts with the distal duplex (14), a potential target for mutational research to reduce off-target effects (Fig. S3). To test whether acetylation of these three lysine residues exerted an effect on Cas9 activity, we next mutated K33, K954, and K1097 into glutamine (Q), which mimicked lysine acetylation with a neutral charge, to investigate the possible effect of acetylation (using mutants of K-R as controls). As shown in Fig. 1g, Cas9^K33Q^ and Cas9^K1097Q^ almost lost cleavage activity, indicating that the K33 and K1097 acetylation played an important role in the enzymatic activity of Cas9.

We further expanded this assay to other acylation processes, such as succinylation mediated by intracellular succinyl-CoA. WB analysis showed that the Cas9 succinylation level depended on the incubation time and succinyl-CoA concentration (Fig. 1h and i). The cleavage activity decreased gradually as the succinyl-CoA concentration increased (Fig. 1j). These results demonstrated that AcP or acyl-CoAs can ac(et)ylate Cas9 enzyme in vitro and inhibit Cas9 cleavage activity.

### Acetylation of *Spy*Cas9 decreased the binding and cleavage activity *in vivo*

**Fig. 2.**
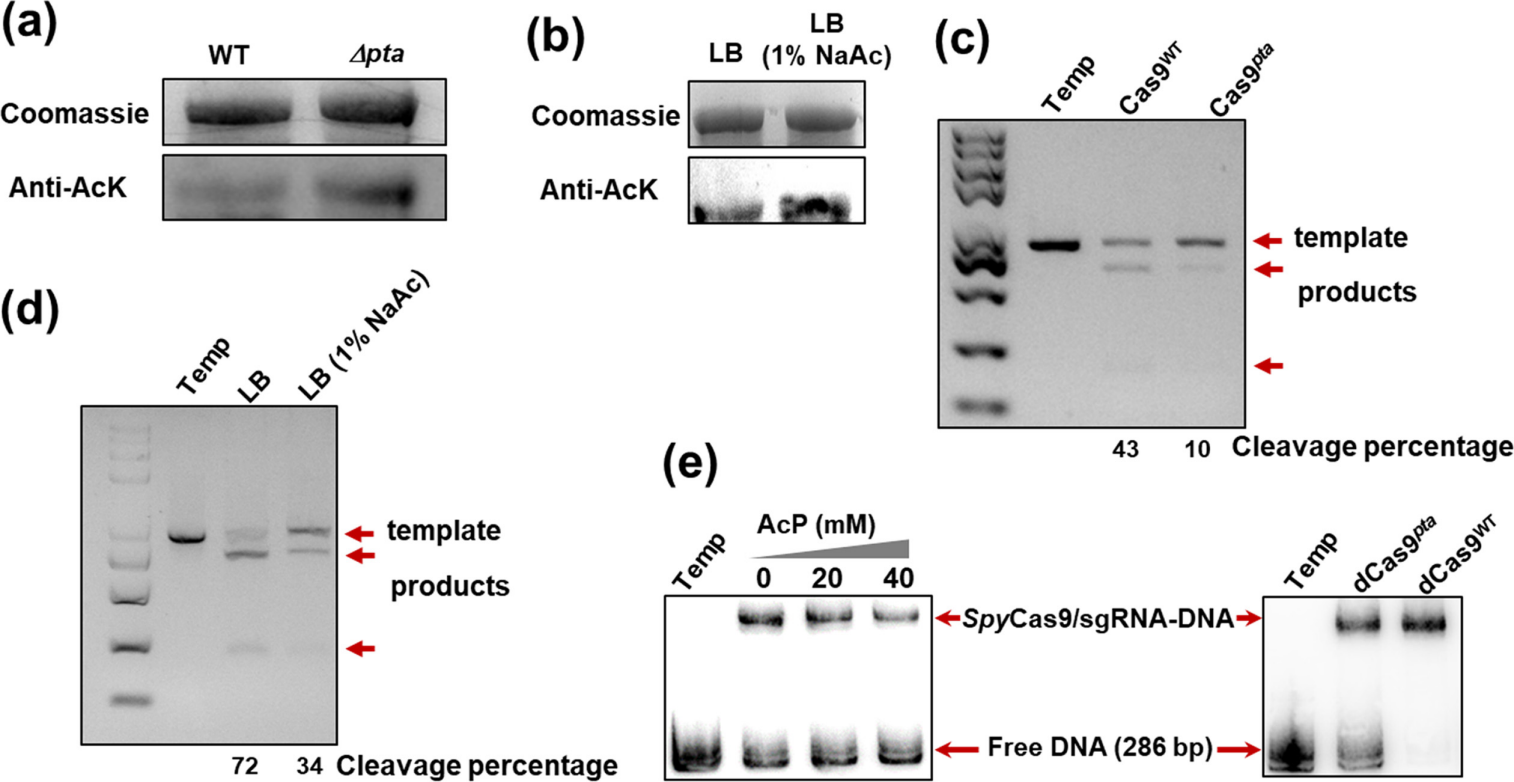
Acetylation of *Spy*Cas9 decreased the binding and cleavage activity *in vivo*. (a) *E. coli* MG1655 (WT) and *Δpta* mutant were growth-arrested in LB. Cas9 proteins were isolated from both strains, and the acetylation was analyzed by using anti-AcK. (b) WT strain was cultured in LB and LB supplemented with 1% NaAc, respectively. Cas9 proteins were isolated, and the acetylation was analyzed using anti-AcK. (c) Cleavage efficiency was measured using the Cas9 proteins that were purified from the WT and Δ*pta* strains. (d) Cutting efficiency was measured using the Cas9 that was purified from WT strain cultured in LB and LB supplemented with 1% NaAc. (e) Binding inhibition assays were conducted by using dCas9 acetylated with increasing concentrations of AcP (0, 20, and 40 mM) and dCas9 proteins purified from WT and Δ*pta*.

To confirm that AcP also acetylates *Spy*Cas9 in vivo, we constructed an *E. coli* Δ*pta* strain (phosphotransacetylase-deleted strain), which had a high concentration of intracellular AcP and a high level of protein acetylation (Fig. S4a) (12). We found that the acetylation level of Cas9 introduced in the Δ*pta* strain obviously increased compared to that in the WT (*E. coli* K-12 MG1655) strain (Fig. 2a). Addition of NaAc (1%) also resulted in the accumulation of intracellular AcP in the WT strain (Fig. S4b) and an increase in the Cas9 acetylation level (Fig. 2b). Cas9 isolated from *E. coli* was analyzed by Nano-HPLC-MS/MS, and many acetylated sites were identified, including K33, K954, and K1097. To examine the effect of Cas9 acetylation levels on Cas9 activity in *E. coli*, Cas9 enzymes were overproduced and purified from *E. coli* cells (Δ*pta* strain, WT strains with or without 1% NaAc). Equal amounts of purified Cas9 were used for cleavage assays. The results showed that the cleavage activity of Cas9 purified from Δ*pta* and WT (1% NaAc) strains was significantly lower than that of the wild-type strains (Fig. 2c,and d). An electrophoretic mobility shift assay revealed that AcP-acetylated Cas9 and dCas9 from the Δ*pta* strain exhibited weak binding of sgRNA-guided *Spy*Cas9 with DNA (Fig. 2e). These observations indicated that Cas9 was acetylated in host cells, leading to decreased cleavage activity and binding affinity.

### Cas9 acetylation exerted an effect on genome editing efficiency in host cells

**Fig. 3.**
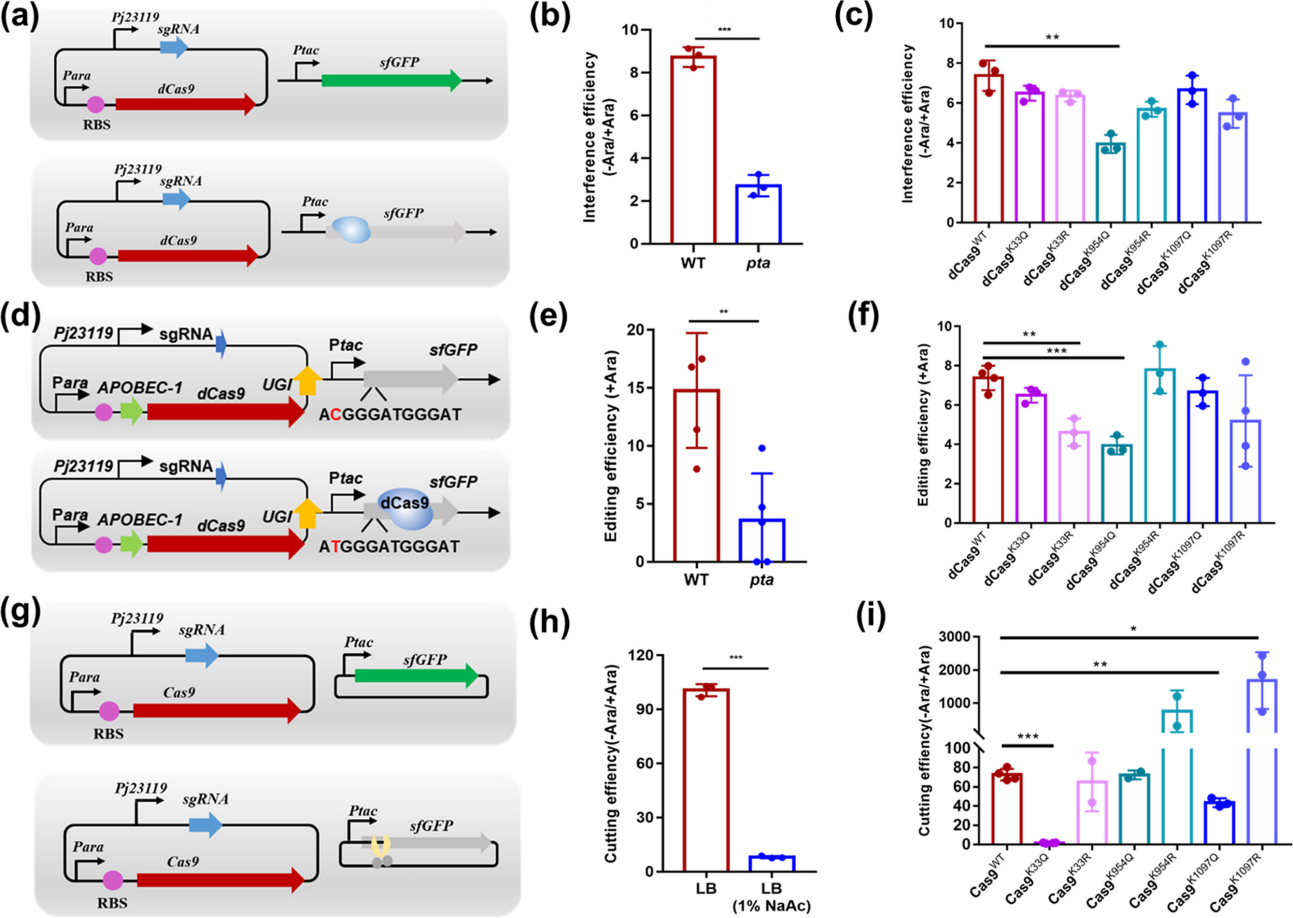
The effect of Cas9 acetylation on genome editing in host cells. (a) Overview of the dCas9-sfGFP interference system used to investigate the binding activity of dCas9 with dsDNA (gene silencing). (b) The interference efficiencies were measured in Δ*pta* and WT strains cultured in LB by using the dCas9-sfGFP interference system. (c) The interference efficiencies of acetyl-mimetic mutants and anti-acetyl-mimetic mutants were measured using the dCas9-sfGFP interference system. (d) Overview of the dCas9-sfGFP editing system used to investigate the binding activity of dCas9 with dsDNA (translation activation). (e) Editing efficiencies were measured in Δ*pta* and WT strains cultured in LB by using the dCas9-sfGFP editing system. (f) The editing efficiencies of acetyl-mimetic mutants and anti-acetyl-mimetic mutants were measured using the dCas9-sfGFP editing system. (g) Overview of the Cas9-sfGFP cutting system used to investigate the dsDNA cleavage activity of Cas9 (gene knockout). (h) Cleavage efficiencies were measured in *Δpta* and WT strains cultured in LB using the Cas9-sfGFP cutting system. (i) Cleavage efficiencies of acetyl-mimetic mutants and anti-acetyl-mimetic mutants were measured using the Cas9-sfGFP cutting system.

To explore the effect of Cas9 acetylation in host cells on the binding activity of dCas9 with dsDNA (gene silencing), we developed a dCas9-*sfGFP*-based interference system in which the inactive dCas9 (with arabinose-induced promoter) binds but does not cleave the target region (the 39th base to the 58th base) of the *sfGFP* fluorescence gene, thereby blocking its transcription in *E. coli* (Fig. 3a). The results showed that dCas9 revealed 8.7-fold repression on *sfGFP* expression in the WT strain. The acetylation of dCas9 relieved this repression (only 2.7-fold) in the Δ*pta* strain, indicating that its binding with the *sfGFP* gene was inhibited by up to three times (Fig. 3b). The dCas9 acetyl-mimetic mutants (K33Q, K954Q, and K1097Q) and the anti-acetyl-mimetic mutants (K33R, K954R, and K1097R) were also tested in this system. dCas9^K954Q^ significantly decreased the repression of sfGFP expression (Fig. 3c) and dCas9^K954R^ rescued the decreased inhibition. Furthermore, a single-base editing system (C-T), in which the endogenous initiation codon and code the methionine (ATG) of *sfGFP* was replaced with an editable ACG-tag sequence (silencing the sfGFP translation), was used to investigate the effect of dCas9 acetylation on editing efficiency (13,14) (Fig. 3d). It was found that dCas9, with high acetylation levels in the Δ*pta* strain, and dCas9^K954Q^ substantially inhibited the single-base editing efficiency by 4-fold and 4.9-fold, respectively; and dCas9^K954R^ rescued the single-base editing efficiency (Fig. 3e and f). We next tested the effect of Cas9 acetylation on dsDNA cleavage activity (gene knockout). Similarly, a Cas9-*sfGFP*-based knock-out system was developed, in which the Cas9 (with arabinose-induced promoter) cuts the *sfGFP* gene (Fig. 3g). As shown in Fig. 3h, arabinose-induced expression of Cas9 in *E. coli* cultured in LB has approximately 12.5-fold cutting efficiency of the *sfGFP* gene than that observed in LB with 1% NaAc. We also compared the activity of the three acetyl-mimetic mutants and anti-acetyl-mimetic mutants to WT Cas9 to determine the cleavage efficiency of the gene in *E. coli*. The data showed that Cas9^K33Q^ and Cas9^K1097Q^ significantly reduced cleavage efficiency by at least 42-fold for Cas9^K33Q^ and 1.5-fold for Cas9^K1097Q^, respectively; Cas9^K33R^ showed similar cleavage efficiency with WT Cas9, and Cas9^K1097R^ displayed obviously higher cleavage efficiency than WT Cas9 (Fig. 3i). The *in vitro* and *in vivo* experimental results (Fig. 1g and Fig. 3h and i) demonstrated that the positive charge of two sites (K33 and K1097) interacting with the sgRNA directly (13) played a key role for target gene cleavage, and K-R mutation of K1097 and K954 significantly improved the cleavage activity of Cas9. On the other hand, the RuvC loop that carries a patch of positively charged residues (K948, R951, and K954), which interact with the distal duplex, could be important for binding of Cas9 with the target gene (14). In this study, we found that the introduction of a neutral charge (K954Q) indeed significantly decreased binding activity of dCas9 with target gene (Fig. 3c and f). Considered together, these results suggest that metabolite-driven acetylation of Cas9 in host cells had inhibitory effects on Cas9 activity and the genome editing application of CRISPR in vivo: blocking the binding of dCas9 to target DNA and impeding its function as a transcriptional repressor or base editor; causing a decrease of Cas9 cleavage activity unable to cut gene. And Cas9^K1097R^ may serve as a potential genome editing tool in the future.

### Acetylation of cas12a decreased the *cis*- and *trans*-cleavage activities *in vitro*.

**Fig. 4.**
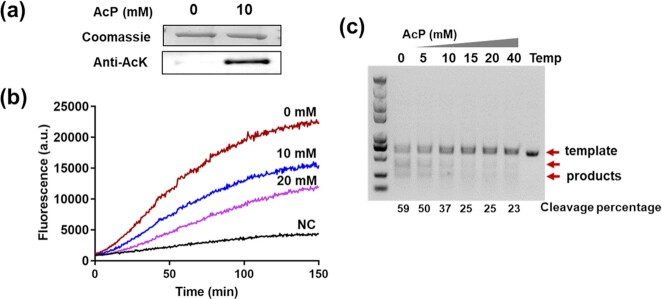
Cas12a acetylation decreased the *cis*- and *trans*-cleavage activities*in vitro*. (a) *Lb*Cas12a was incubated with or without 10 mM AcP at 37°C for 3 h *in vitro*. Products were assessed by SDS-polyacrylamide gel electrophoresis and the acetylation levels were analyzed by Western blotting using the Anti-AcK antibody. (b) AcP-induced *trans*-cleavage inhibition assays on ssDNA by using *Lb*Cas12a treated with 0, 10, and 20 mM AcP. (c) AcP-induced *cis*-cleavage inhibition assays on dsDNA by using *Lb*Cas12a treated with 0, 5, 10, 15, 20, and 40 mM AcP. NC was the treatment without dsDNA target added.

Lastly, Cas12a was used to investigate the effect of acetylation on other Cas proteins. We found that Cas12a could be acetylated when incubated with 10 mM AcP at 37°C for 3 h (Fig. 4a), and the *trans*-cleavage activities and *cis*-cleavage activities of Cas12a decreased gradually with increasing AcP concentration (Fig. 4b, 4c). These observations indicate that acetylation can regulate other Cas enzymes.

In summary, to our knowledge, this is the first study to demonstrate that ac(et)ylation of Cas enzyme driven by intracellular acetyl-phosphate or acyl-CoA metabolites in host cells inhibited its binding and cleavage activity with target nucleic acids, thereby decreasing the genome editing efficiency of CRISPR. This anti-crispr mechanism based on nonenzymatic acylation functions in a distinct manner from various Acr-mediated mechanisms that phages have developed and is likely an efficient strategy to effectively evade CRISPR immune systems. It will be interesting to investigate whether phages employ intracellular high-energy metabolites in hosts to covalently modify the Cas proteins, thus inhibiting CRISPR-mediated immunity (the phage infection can result in an increase in intracellular levels of AcP and acyl-CoA in hosts?). More importantly, our study provides additional insight into the effects of intracellular environments (esp. metabolic status or shift) in the genome editing application of CRISPR in host cells, what's more, we found an anti-acetyl-mimetic mutant, Cas9^K1097R^, which may serve as a potential genome editing tool in the future.

## Methods

### Bacterial culture

*E. coli* K-12 MG1655 (WT) and *Δpta* strains were grown in lysogeny broth (LB) medium or in LB with 1% (w/v) or 0.27% (w/v) acetate sodium based on the experimental needs.

### Protein expression and purification

The His-SpyCas9 recombinant protein was induced by adding 0.5 mM IPTG at 18°C when OD reached to 0.6. After overnight induction, cells were collected by centrifugation at 4000 g for 10 min and washed once in 1 x PBS. After resuspended in buffer 1 x PBS, the cells were lysed by sonication and supernatants were harvested by centrifugation at 9000 g for 10 min at 4°C. (d)Cas9 proteins were purified by the Ni-NTA agarose resin (TransGen Biotech). First, the Ni-NTA agarose column preloaded was equilibrated with 10 mL Buffer A (10 mM imidazole, 50 mM NaH_2_PO4, 300 mM NaCl, pH 8.0). Second, supernatants from the lysates were flowed through the column, and 20 mL Buffer B (20 mM imidazole, 50 mM NaH_2_PO_4_, 300 mM NaCl, pH 8.0) were added to remove the other proteins. Third, 20 mL Buffer C (50 mM imidazole, 50 mM NaH_2_PO_4_, 300 mM NaCl, pH 8.0) were added to further remove other protein. Finally, (d)Cas9 protein was obtained by eluted by 5 mL Buffer D (250 mM imidazole, 50 mM NaH_2_PO_4_, 300 mM NaCl, pH 8.0). Amicon Ultra Centrifugal Filters (UFC9030) centrifugal tube was used to concentrate Cas9 protein. Cas9 proteins were identified by Coomassie-stained SDS-PAGE analysis, and protein concentration was determined by BCA reagents with BSA as the standard.

Plamid pET28a-LbCas12a was lab-preserved and transformed into BL21(DE3). The purification method was slightly different from Cas9. After LbCas12a was induction, cells were resuspended in buffer E (10 mM imidazole, 50 mM Tris-HCl, 1.5 M NaCl, 1 mM DTT, pH 8.0), lysed by sonication and supernatants were harvested. After equilibrating with buffer E, proteins were loaded on the Ni-NTA agarose resin. Buffer F (30 mM imidazole, 50 mM Tris-HCl, 1.5 M NaCl, 1 mM DTT, pH 8.0) was added to wash the other proteins. Then, Buffer G (600 mM imidazole, 50 mM Tris-HCl, 1.5 M NaCl, 1 mM DTT, pH 8.0) was used to elute LbCas12a protein. Finally, Buffer H (50 mM Tris-HCl, 200 mM NaCl, 1 mM DTT, 5% glycerol, pH 8.0) was used as a stock solution.

### *In vitro* transcription and purification of sgRNA

DNA templates EMX1(1) of sgRNA with T7 promoter was amplified from the genome of HEK293 cells using primers EMX1(1) gRNA (5′- TTAATACGACTCACTATAGGGAGTCCGAGCAGAAGAAGAAGTTTT AGAGCTAGAAATAGC-3′) and gRNA-reverse (5′-agcctcagtcttcccatcagg-3′). HiScribe T7 Quick High Yield RNA Synthesis Kit (NEB) was used to transcribe DNA to RNA. 10 μL NTP buffer mix (5 mM each NTP), 1 μg DNA templates, 2 μL T7 RNA polymerase mix and appropriate DEPC H_2_O were added to 20 μL transcription reaction mixture. The transcription products were harvested after incubation at 37°C overnight, purified by a HiPure RNA Pure Micro Kit (Magen) and stored at −80°C. The concentration of the purified sgRNAs was determined with a NanoDrop 2000 apparatus.

### *In vitro* cleavage assay

DNA templates EMX1(1) fragment for cleavage activity was amplified from the genome of HEK293 cells using primers EMX1(1)-F (5′-agcctcagtcttcccatcagg-3′) and EMX1(1)-R (5′-tgaccccctccaccagtacc-3′). The cleavage experiment was performed in 20 μL 1 × NEB Buffer 3.1 system containing 100 nM spyCas9, 200 nM sgRNA, 100 ng EMX1(1) fragment and 1µL proteinase K. First, 1 × NEB Buffer 3.1 (100 mM NaCl, 50 mM Tris-HCl, 10 mM MgCl_2_, 100 µg/ml BSA, pH 7.9), 100 nM spyCas9, 200 nM sgRNA were incubated together at 37°C for 10 min to form sgRNA: Cas9. Then, 100 ng EMX1(1) fragment were added to above system and were incubated at 37°C for 1 h. After that, 1 μL Proteinase K were added to the 20 μL reaction system quenched at 85°C for 6 min to terminate the reaction. Cleavage products were separated on 2% agarose gel electrophoresis. The percentage of cleavage was calculated based on the gray levels determined by Image J. The computational formula of cleavage was $R(\%) = \frac{\rm{products}}{\rm{products + left\,\, template}} \times 100$.

For AcP-mediated inhibition of dsDNA cleavage by spyCas9, 100 nM spyCas9 proteins were first incubated with 0, 5, 10, 15, 20, and 40 mM AcP in cleavage buffer 100 mM NaCl, 50 mM Tris-HCl, 10 mM MgCl_2_, pH 7.9) at room temperature for 20 min. Then, 200 nM sgRNA was added to the above cleavage mixture and incubated at 37°C for 10 min. After that, 100 ng EMX1(1) fragment was added to the reaction mixture and incubated at 37°C for 1 h. Finally, 1 μL Proteinase K were added to the 20 μL reaction system and quenched at 85°C for 6 min to terminate the reaction. Cleavage products were analyzed on 2% agarose gel electrophoresis and the percentage of cleavage was determined by the computational formula above.

For succinyl-CoA-mediated inhibition of dsDNA cleavage by spyCas9, 100 nM spyCas9 proteins were first incubated with 0, 20, 50, 250, 500 μM succinyl CoA in cleavage buffer at room temperature for 20 min. And next steps are as same as AcP-mediated inhibition of dsDNA cleavage assay. And AcP-mediated inhibition *cis*-cleavage activities on dsDNA by LbCas12a was performed according to this method.

For AcP-mediated-inhibition trans-cleavage activities on ssDNA (FAM-TTATT-BHQ) by LbCas12a, 100 nM LbCas12a proteins were first incubated with 0, 10, and 20 mM AcP in cleavage buffer at room temperature for 20 min. Then 100 ng FimA fragment and 1 μL 20 μM ssDNA were added to the cleavage mixture. Instantly, the reaction mixture was moved to the black 384-well plates (Fluotrac 200; Greiner, Germany) and the fluorescence was detected by a microplate reader (BioTek Instrument, Winooski, VT, USA).

### Electrophoretic mobility shift assay

Electrophoretic mobility shift assays (EMSAs) were performed using catalytically inactive dCas9 proteins purified from WT and Δ*pta*. The biotin-modified target dsDNA fragment was first amplified using primers 286bp-EMX1 (1)-F (5'-AGCCAGTGGCGATAAGCCATCAGGCTCTCAGCTCAG-3′) and 286bp-EMX1 (1)-R (5′-AGCCAGTGGCGATAAGgtgggtttgtggttgcccac-3′). The biotin-modified universal primer 5'-biotin-AGCCAGTGGCGATAAG-3' was used to amplify the biotin-modified fragment.

For the gel shift assay with dsDNA binding, 100 nM dCas9 proteins purified from WT and Δ*pta* and 200 nM sgRNA were added to 1 × NEB Buffer 3.1 and incubated at 37°C for 10 min. Then, 100 ng of 5′ biotin-modified dsDNA was added to the above 10 μL system and incubated at 37°C for 30 min. The DNA-protein complexes were analyzed at 100 V on a 6% (w/v) non-denaturing Tris-glycine–polyacrylamide gel (29:1 Acrylamide/bisacrylamide) in 0.5 × TBE buffer. EMSAs were performed using the Chemiluminescent EMSA Kit (Beyotime Biotechnology, China).

For AcP-mediated inhibition of dsDNA binding by dCas9, 100 nM dCas9 proteins were first incubated with 0, 20, and 40 mM AcP in cleavage buffer at room temperature for 20 min. Then, 200 nM sgRNA was added to the above binding mixture and incubated at room temperature for 10 min. Next, 5′ biotin-modified dsDNA was added to the reaction mixture and incubated at 37°C for 1 h. Finally, the samples were run on PAGE gels, and EMSA was performed.

### *In vivo* cleavage assay

Plasmids pWT-*Ara*-*Cas9* and pProBE-*sfGFP* were constructed and co-transformed into DH5α competent cells following the manufacturer's protocol to explore the inhibitory effects of AcP on spyCas9-mediated dsDNA cleavage. The transformation was recovered at 37°C for 2 h and was spread on LB agar plates with streptomycin (50 μg mL^–1^) and tetracycline (50Īμg mL^–1^). After incubation at 37°C for 12 h, three monoclonal colonies were chosen for culture at 37°C for 12 h for further use. DH5α cultured in LB supplemented with 1% NaAc was regarded as an AcP-accumulated acylation environment. 4μL bacterial suspension of one monoclonal colony were added to 800 μL LB (0.2% Ara added and without added) and 800 μL LB supplemented with 1% NaAc (0.2% Ara added and without added) individually in a 24-well plate. After cultivation at 37°C for 12 h, 600 μL of the bacterial suspension was collected and used for qPCR analysis. qPCR was used to determine the transcriptional levels of *sfGFP* and *16S rRNA* was defined as internal reference. The transcriptional levels of *sfGFP* were normalized to the levels of the *16S rRNA* transcript. The cutting efficiency was calculated by the calculation formula: “Cutting efficiency (-Ara/+Ara)” $= \frac{\rm{sfGFP \,\, transcriptional \,\, levels \,\, in \,\, samples \,\, without \,\, Ara}}{\rm{sfGFP \,\, transcriptional \,\, levels \,\, in \,\, samples \,\, with \,\, Ara}}$.

### *In vivo* interference assay and single-base editing assay

Interference assays and single-base editing assays were performed to determine the binding activity of dCas9 and target dsDNA, interference assay and single-base editing assay were performed. For the interference assay, GI22-WT and GI22-Δ*pta* strains were constructed. GI22-WT was a strain with the reporter gene*sfGFP* driven by the tac promoter integrated in the genome of WT. GI22-*Δpta* was a strain with the *pta* gene deleted on the foundation of GI22-WT using homologous recombination based on the λ-Red system. The pta-F (5′-ggcagagcagatcatctctg-3′) and pta-R (5′-tccggttcagatatccgcag-3′) were used to identify whether the pta gene was successfully deleted. Plasmid pWT-*Ara*-*dCas9* was individually transformed into GI22-MG1655 and GI22-*Δpta*. Primers cx-33R (5′-ttttctggaagttgttgtcg-3′) and primer pENTR1A-3F (5′-gtaacatcagagattttgagacac-3′) were used to identify whether the transformation was successful. Three monoclonal colonies were chosen for culturing at 37°C overnight. 4 μL pWT-*Ara*-*dCas9*/GI22-WT bacterial suspension was added to 800 μL LB and 4 μL pWT-*Ara*-*dCas9*/GI22-*Δpta* bacterial suspension was added to 800 μL LB supplemented with 0.27% NaAc individually in 24-well plates. Initially, 0.2% Ara was added, and the bacterial suspensions were cultured at 37°C for 12 h. When dCas9 was induced by 0.2% Ara, ratio of *sfGFP* fluorescence with OD_600_ decreased when compared to the samples without Ara. The reduced ratio was regarded as binding activity. The interference efficiency was calculated by the calculation formula: “Interference efficiency (-Ara/+Ara)” $= \frac{\rm{sfGFP \,\, fluorescence/OD_{600} \,\, of \,\, samples \,\, without \,\, Ara}}{\rm{sfGFP \,\, fluorescence/OD_{600} \,\, of \,\, samples \,\, with \,\, Ara}}$. In addition, all mutated plasmids pWT-*Ara*-*dCas9*^K33Q/R^, pWT-*Ara*-*dCas9*^K954Q/R^, and pWT-*Ara*-*dCas9*^K1097Q/R^ were transformed into GI22-WT and the interference efficiency was calculated according to the calculation formula.

For the single-base editing assay, GO5-WT and GO5-*Δpta* strains were constructed. pWT-*Ara*-*BE2* contains a pSC101 replication origin, an arabinose (Ara) inducible a second-generation base editor BE2 (APOBEC-XTEN-dCas9-UGI) and a constitutive promoter P_j23119_ regulated sgRNA2 (5'-tgaacgggatgggatagctg-3'). GO5-WT was a strain with a modified reporter gene *sfGFP* driven by the tac promoter integrated into the genome of WT. GO5-*Δpta* was a strain with the *pta* gene deleted on the foundation of GO5-WT using homologous recombination based on the λ-Red system. Plasmid pWT-*Ara*-*BE2* was transformed into GO5-WT and GO5-*Δpta*. When dCas9 was induced by 0.2% Ara, dCas9 could bind to the genomic DNA, and BE2 converted cytosine (C) to thymine (T). The efficiency of the conversion represents the binding activity. Similar to the interference assay, all the mutated plasmids pWT-*Ara*-*BE2*^K33Q/R^, pWT-*Ara*-*BE2*^K954Q/R^, and pWT-*Ara*-*BE2*^K1097Q/R^ were obtained with 10 ng pWT-*Ara*-*BE2* as the amplified template. They were transformed into GO5-WT and sequencing after PCR was done in a 50 μL system using primers (amplified primers were 5′-GGTGTATATGGCGAGCGCAAT-3′ and 5′-caggatattgccgtcttctttaaag-3′ and sequencing primer was 5′-GGCGGACTTGAAGAAGTCAT-3′) and using the bacteria with Ara added as template. The chromatogram obtained by Sequalizer analysis (MATLAB R2018a) was used to calculate the base-editing rate at the second position of the *sfGFP*gene.

### AcP measurement

The content of AcP was measured according to the procedure as described ^(1)^. pProEX-THb-*Cas9*/WT and pProEX-THb-*Cas9*/*Δpta* were cultured in LB medium supplemented with 0.27% NaAc. After induction with 0.5 mM IPTG for 16 h, the bacterial suspensions were harvested by centrifugation at 3000 g for 10 min. The cell pellets were resuspended in ice-cold assay buffer containing 10 mM sodium phosphate (pH 7.5), 10 mM MgCl_2_, 1 mM EDTA, and diluted to OD_600_ 0.5. Next, 125 μL ice-cold 3 M HClO_4_ was added to 500 μL bacterial suspensions and incubated on ice for 30 min. The supernatant (500 μL) was harvested by centrifugation at the maximum speed of revolution for 10 min. To neutralize redundant HClO_4_, 125 μL saturated KHCO_3_ was slowly added to the supernatant. When there were no air bubbles, the above reaction mixture was centrifuged, and 500 μL of neutralization liquid was collected. 37.5 mg powdered activated charcoal (Sigma) was added to the neutralization liquid to remove intrinsic ATP, incubated on ice for 15 min, and filtrated through a 0.45 μm spin filtration to remove the charcoal. 1 μL 100 mM MgCl_2_, 6 μL 1 mM ADP (Cell Technology), and 4 μL 0.4 μg/μL acetate kinase (Sigma) and 200 μL filtered fluid were added to 100 mM Triethanolamine buffer to convert ADP to ATP and incubated at 30°C for 90 min. A 100 μL  onversion reaction was mixed with 100 μL CellTiter‐Glo (Promega) reagent and incubated at 25°C for 10 min. Luminescence was measured using a microplate reader in a white 96-well microplate. The standard curve of ATP is plotted.

### Real-time qRT-PCR analyses

Total RNA from the bacteria was extracted using a RNAprep pure Cell/Bacteria Kit (DP430; Tiangen), and cDNAs were synthesized using TransScript Uni All-in-One First-Strand cDNA Synthesis SuperMix for qPCR (One-Step gDNA Removal; AU341-02; Transgen) according to the manufacturer's instructions. Before qPCR, the 10 μL reverse transcription products were diluted 10-fold with DEPC H_2_O, which was performed by a Bio-Rad CFX 96 real-time PCR detection system (Bio-Rad, Hercules, CA, USA) using the PerfectStart Green qPCR SuperMix (AQ601-02; Transgen). Forward and reverse sfGFP primers are sfGFP-F (5′-gttcactggtgtcgtcccta-3′) and sfGFP-R (5′-gccaaggtaccggcagttta-3′). qPCR data were analyzed with the CFX software version 1.1. 16S rRNA was used as an internal control and the primers are 16S rRNA-F (5′-TCGCGTTGCATCGAATTAAA-3′) and 16S rRNA-R (5′-CCCCCTGGACGAAGACTGAC-3′). All PCR reactions mixture were prepared in a volume of 20 µL for triplicate and followed the manufacturer's instructions recommended protocols.

### Western blot

(d)Cas9 proteins purified from MG1655 were mixed with 6 × protein loading buffer and resolved by SDS-PAGE and transferred to a PVDF membrane (Merck, Millipore) at 380 mA for 90 min. After washed with TBST buffer (20 mM Tris-HCl, pH 7.6, 150 mM NaCl, and 0.1% (v/v) Tween 20) twice, PVDF membrane was incubated with 10 mL 0.5% BSA blocking buffer at room temperature for 2 h. 2 µL anti-acetyl-lysine antibody (Anti-AcK; Cell Signaling Technology) or succinylated antibody (Anti-SucK; Cell Signaling Technology) was added to the BSA blocking buffer and incubated at room temperature for 1.5 h or incubated at 4 °C overnight. Then the PVDF membrane was washed by TBST buffer for three times and 2 µL ProteinFind Goat Anti-Mouse IgG (H + L) (HRP Conjugate; HS201-01; Transgen) was diluted to 10 mL TBST buffer. After incubation for 55 min and then washing for three times using TBST buffer, the ECL system (CTB, USA) was used to detect the signal according to the manufacturer.

### Protein extraction and tryptic digestion

Protein (10 μg) prepared for identification of the acetylation site was run on 10% SDS-PAGE, and the target protein was collected after staining. The proteins were digested according to the filter-aided sample preparation (FASP) procedure described by Wisniewski et al. (15) First, 10 μL SDT buffer (4% SDS, 100 mM DTT, and 150 mM Tris-HCl pH 8.0) was prepared, and the protein was solubilized at 90°C for 5 min. Second, the UA buffer (8 M urea, 150 mM Tris-HCl, pH 8.0) was used to wash the SDT buffer by multiple ultrafiltration (Microcon units, 50 kD), and 50 μL 0.05 M iodoacetamide was added to the ultrafiltration product to block the reduced cysteine residues and incubated for 30 min in the dark. Third, UA buffer and 25 mM NH_4_HCO_3_ were successively used to wash the filter. Fourth, the protein was suspended in 40 μL 25 mM NH_4_HCO_3_ containing 2 μg trypsin (Promega) and incubated for 12 h at 37°C. Finally, the resulting peptides were harvested as filtrates.

### Nano-HPLC-MS/MS analysis

The peptides were identified using mass spectrometry according to the procedure as described (16). MS/MS spectra were searched using the MASCOT engine (Matrix Science, London, UK; version 2.2) against Uniprot *S. pyogenes* serotype M1 database. Protein identification options are as followings. Peptide mass tolerance = 20 ppm, Nano-HPLC-MS/MS tolerance = 0.1 Da, Enzyme = Trypsin, Missed cleavage = 2, Fixed modification: Carbamidomethyl (C), Variable modification: oxidation (M), acetylation (K, N-terminal). All acquired data were based on 99% confidence for spyCas9 protein identification, as determined by false discovery rate (FDR) ≤ 1%.

## Supplementary Material

pgac277_Supplemental_File

## Data Availability

All study data are included in the article and/or SI Appendix.
