# Supplementary material for: Acylation driven by intracellular metabolites in host cells inhibits Cas9 activity used for genome editing
Source: PNAS Nexus. 2022 Dec 6;1(5):pgac277. doi: 10.1093/pnasnexus/pgac277 (PMC9802096; doi:10.1093/pnasnexus/pgac277)
Supplement: pgac277_Supplemental_File [file pgac277_supplemental_file.docx]

**Supporting Information**

**Acylation driven by intracellular metabolites in host cells inhibits Cas9 activity used for genome editing**

Li Zhao^a^, Di You^a^, Ting Wang^a^, Zhen-Ping Zou^a^, Bin-Cheng Yin^a, b^, Ying Zhou^a^, Bang-Ce Ye^a, b^

^a^Laboratory of Biosystems and Microanalysis, State Key Laboratory of Bioreactor Engineering, East China University of Science and Technology, Shanghai 200237, China

^b^Institute of Engineering Biology and Health, Collaborative Innovation Center of Yangtze River Delta Region Green Pharmaceuticals, College of Pharmaceutical Sciences, Zhejiang University of Technology, Hangzhou 310014, Zhejiang, China

**Appendix: Materials and Methods**

**Plasmids construction and site-specific mutagenesis.** Plasmid pWT-Ara-BE2 used in single-base editing system was lab-preserved.

To construct the pPROEX-THb-dCas9 plasmids, pWT-Ara-BE2 was used as the template to amply the gene dCas9 using primers dCas9-F (5′-gataagaaatactcaataggcttagctatcg-3′) and dCas9-R (5′-gtcacctcctagctgactcaaatcaatg-3′). dCas9 was inserted the protein expression vector pPROEX-THb amplied by pPROEX-THb-F (5′-tgatttgagtcagctaggaggtgacAAGCTTGGCTGTTTTGGCGG-3′) and pPROEX-THb-R (5′-ctaagcctattgagtatttcttatcCATAATTCCGGATCCCATGGCGCCC-3′) to form pPROEX-THb-dCas9. To obtain plasmid pPROEX-THb-Cas9, Fast Mutagenesis System (Transgen) was used. 12.5 μL 2 x TransStart FastPfu Fly PCR SuperMix, 0.5 μL K10D-F (10 μM, 5′-ACTCAATAGGCTTAGACATCGGCACAA-3′) and 0.5 μL K10D-R (10 μM, 5′-GTCTAAGCCTATTGAGTATTTCTTAT-3′) and 10 ng pPROEX-THb-dCas9 were added to the 25 μL PCR reaction system and the PCR reactions was performed using the following protocols: hot start of 94 °C for 5 min, followed by 22 cycles of 94 °C for 20 s, 55 °C for 20 s and 72 °C for 10 min, finally an extension at 72 °C for 10 min. After that, 5 μL of PCR products was run on 1.5% agarose gel electrophoresis to make sure the plasmid was amplified. The remaining 20 μL was added to the 0.5 μL DMT (Transgen) and incubated at 37 °C for 1h. Then product of digestion was transformed into DH5α compenent cells, recovered at 37 °C for 40 min, and spread on the LB agar plates with streptomycin (50 μg/ml). After incubated at 37 °C for 12 h, two monoclonal colonies were added to 5 mL LB and incubation at 37 °C overnight. After plasmid extraction, cx-10A-R (5′-GCGCTAAGGCCAAATAGATTAAG-3′) was used for sequencing. Then, primers K840H-F (5′-GATTATGATGTCGATCACATTGTTCCA-3′) and K840H-R (5′-TGATCGACATCATAATCACTTAAACG-3′) was used to mutate K840A to K840H and plasmid pPROEX-THb-Cas9 was obtained successfully by using primer cx-K954 (5′-GCGGTTGCTTTGCCTATTTC-3′) to sequence. pPROEX-THb-(d)Cas9 plasmids were transformed into WT and *Δpta* individually to induce the proteins expression.

To construct the pWT-Ara-dCas9 plasmids used in the interference system, we quit the editing and repairing components APOBEC-1 and UGI of pWT-Ara-BE2. Mutant pWT-Ara-dCas9^K33Q^ plasmid was constructed using primers K33Q-F (5′-CCGTCTAAAAAGTTCCAGGTTCTGGG-3′) and K33Q-R (5′- GGAACTTTTTAGACGGAACCTTATAT-3′). Mutant pWT-Ara-dCas9^K33R^ plasmid was constructed using primers K33R-F (5′-cgtctaaaaagttcaGggttctggga-3′) and K33R-R (5′-Ctgaactttttagacggaaccttat-3′). Mutant pWT-Ara-dCas9^K954Q^ plasmid was constructed using primers K954Q-F (5′-Ggaactttttagacggaaccttatat-3′) and K954Q-R (5′-Gaacctctcgaataagtttatcattt-3′). Mutant pWT-Ara-dCas9^K954R^ plasmid was constructed using primers K954R-F (5′-ttattcgagaggttaGagtgattacc-3′) and K954R-R (5′- Ctaacctctcgaataagtttatcat-3′). Mutant pWT-Ara-dCas9^K1097Q^ plasmid was constructed using primers K1097Q-F (5′-GTCAATATTGTCAAGCAAACAGAAGT-3′) and K1097Q-R (5′-GCTTGACAATATTGACTTGGGGCATG-3′). Mutant pWT-Ara-dCas9^K1097R^ plasmid was constructed using primers K1097R-F (5′-tcaatattgtcaagaGaacagaagta-3′) and K1097R-R (5′- Ctcttgacaatattgacttggggca-3′).

To construct the K33Q/K33R/K954Q/K954R/K1097Q/K1097R mutants of pWT-Ara-BE2 used in the single-base editing system, primers K33Q-F/R, K33R-F/R, K954Q-F/R, K954R-F/R, K1097Q-F/R and K1097R-F/R were used.

To construct the pWT-Ara-*Cas9* plasmid, template pWT-Ara-d*Cas9* and primers K10D-F/R and K840H-F/R were used. Then, K33Q/K33R/K954Q/K954R/K1097Q/K1097R mutants of pWT-Ara-Cas9 were obtained using primers K33Q-F/R, K33R-F/R, K954Q-F/R, K954R-F/R, K1097Q-F/R and K1097R-F/R.

**Supplemental Figures:**

**Supplemental Figure S1. AcP acetylated K33, K954, and K1097 in *Spy*Cas9.**

**Supplemental Figure S2. Multiple protein sequence alignment of Cas9 orthologs.**

**Supplemental Figure S3.** **Schematic diagram of the sgRNA:target DNA complex.**

**Supplemental Figure S4.** **Intracellular AcP increased the protein acetylation level in *E. coli.***

**Supplemental Figure S5. The full gels of Figure 1c, 1d, 1h, 1i, 2a, 2b, 2e, and 4a.**

**
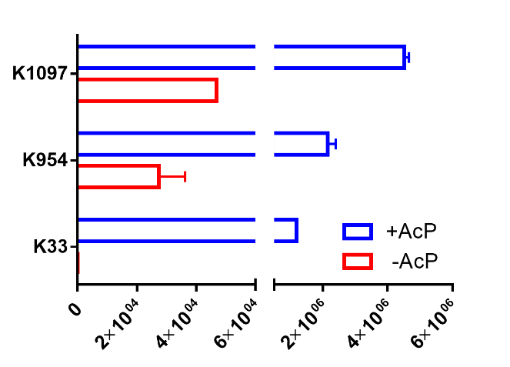
**

**Supplemental Fig. S1** **| AcP acetylated K33, K954, and K1097 in *Spy*Cas9.** Cas9 was incubated with or without 10 mM AcP. The acetylation level of K33, K954, and K1097 was measured by Nano-HPLC-MS/MS semi-quantitative analysis.


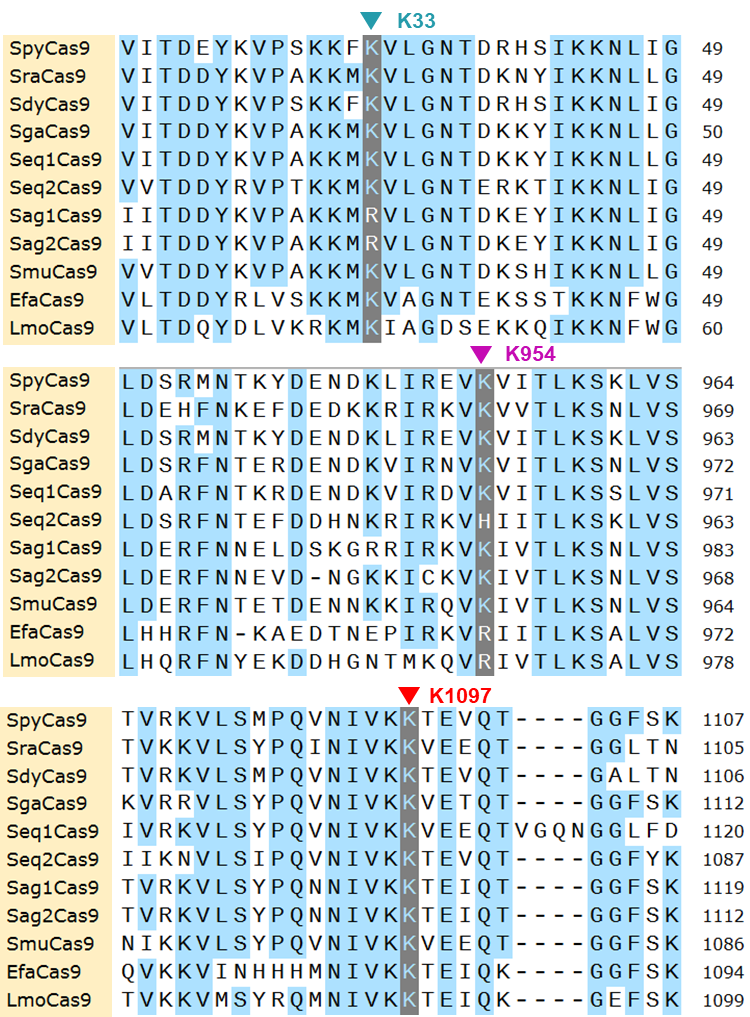


**Supplemental Fig. S2** **| Multiple protein sequence alignment of Cas9 orthologs.** K33, K954, and K1097 were conserved. Sequences of Cas9 proteins derived from *Streptococcus pyogenes*, *Streptococcus ratti*, *Streptococcus dysgalactiae*, *Streptococcus gallolyticus*, *Streptococcus equinus*, *Streptococcus agalactiae*, *Streptococcus mutans*, *Enterococcus faecium,* and *listeria monocytogenes* were compared using Snapgene.


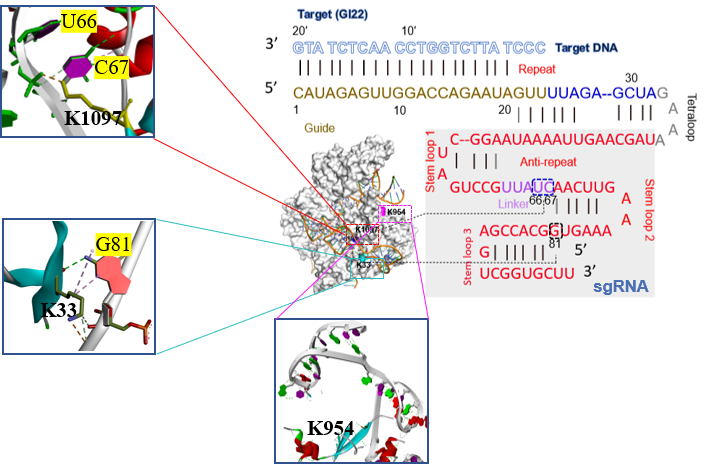


**Supplemental Fig. S3** **|** **Schematic diagram of the sgRNA:target DNA complex.** K33 and K1097 connect with the complex through the main chain. K954 interacts with the distal duplex.


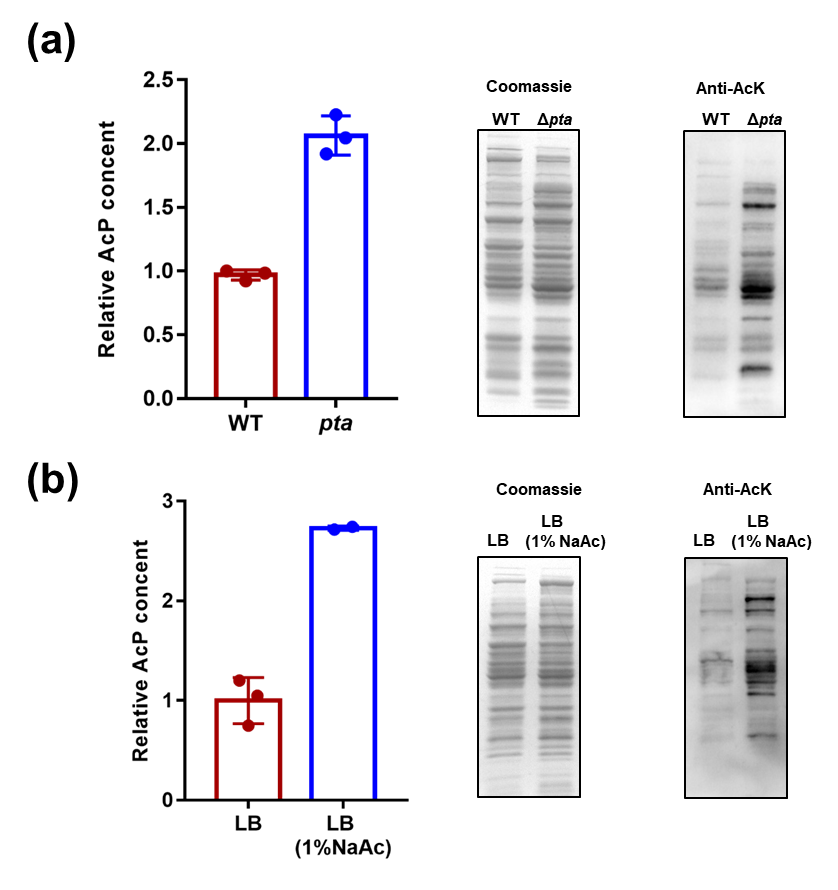


**Supplemental Fig. S4** **|** **Intracellular AcP increased the protein acetylation level in *E. coli*.** (a) The deletion of *pta* gene encoding phosphotransacetylase increased the concentration of intracellular AcP and the protein acetylation level in *E. coli*. (b) Addition of NaAc (1%) resulted in an accumulation of intracellular AcP in WT strain and increased acetylation of proteins in *E. coli*.


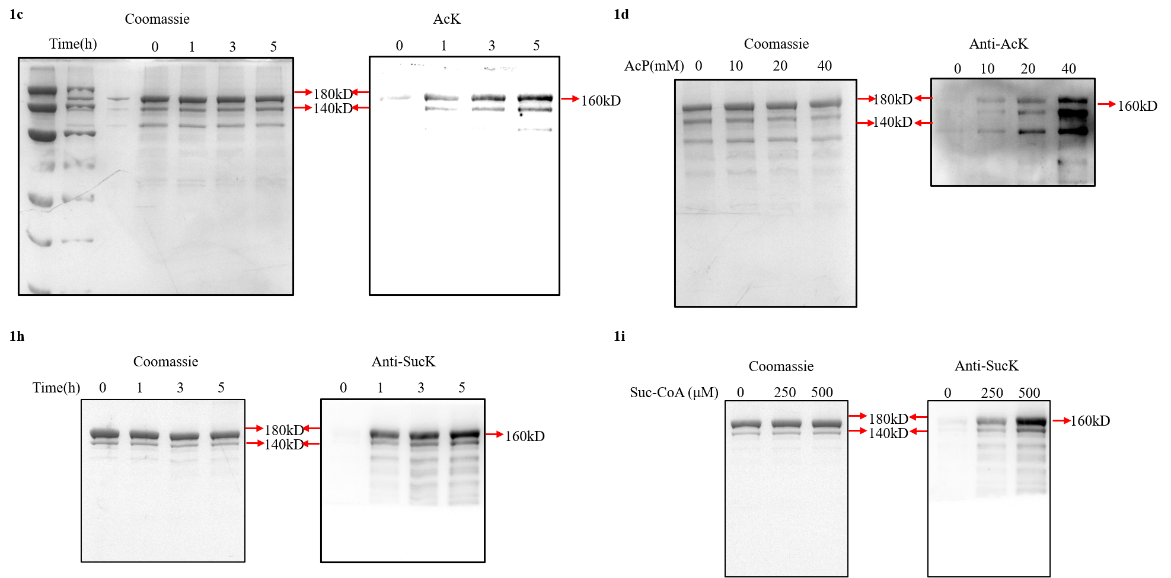


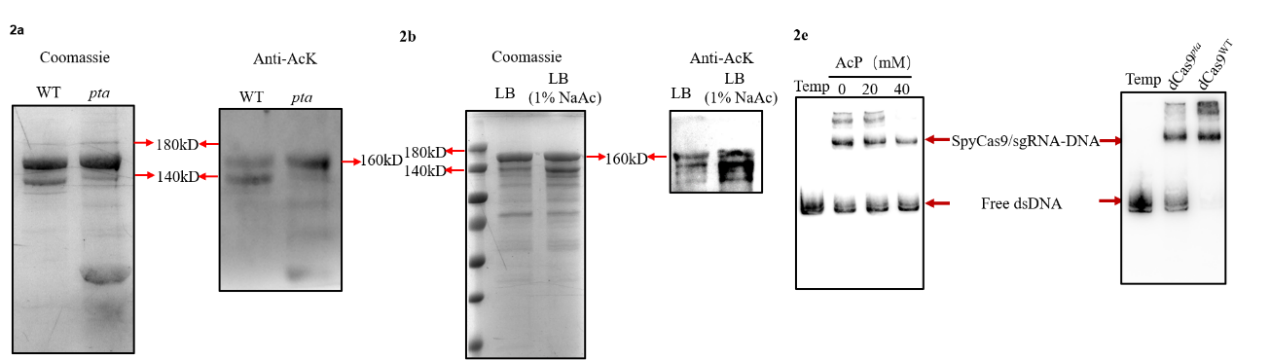


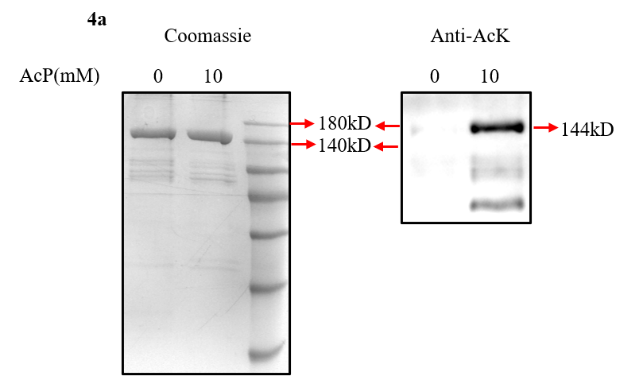


**Supplemental Fig. S5** **|** **The full gels of Figure 1c, 1d, 1h, 1i, 2a, 2b, 2e, and 4a.**
